# Synthesis and utilization of titanium dioxide nano particle (TiO_2_NPs) for photocatalytic degradation of organics

**DOI:** 10.1038/s41598-024-53617-9

**Published:** 2024-05-17

**Authors:** M. G. Kholief, Abd El-Latif Hesham, F. S. Hashem, F. M. Mohamed

**Affiliations:** 1 Faculty of Earth Sciences, Beni-Suef University, P.O. 62521, Beni-Suef, Egypt; 2 Genetics Department, Faculty of Agriculture, Beni-Suef University, Beni-Suef, Egypt; 3 Chemistry Department, Faculty of Science, Ain Shams University, P.O. 11566, Cairo, Egypt

**Keywords:** Environmental chemistry, Environmental impact

## Abstract

A green technique that emerged as a promise in the degradation of numerous organic contaminants is photocatalysis. The aim of this study concerns photocatalytic degradation of organic using titanium dioxide nano particles (TiO_2_ NPs) which syntheses from ilmenite by different leaching methods using different ingredients such as HCl, HNO_3_ and Aqua Regia. The affecting factors such as rate of addition, reaction time, ilmenite grain size, acid to ilmenite ratio and reaction temperature were conducted. Comprehensive physicochemical characterization of Ilmenite and TiO_2_ NPs were conducted using different analytical techniques such as XRD, XRF, SEM, TEM and FTIR. Photocatalytic degradation of organics is confirmed by studies of affecting factors on the effectiveness of TiO_2_ NPs such as dose, agitation forces, light intensity, initial concentration, pH, time, and temperature. The removal percentages of TSS, COD, BOD and TN of organics were explored. From the results the maximum removal percentage of TSS were 97.3 and 96.9% before and after secondary treatment conducted using ferric chloride (FC). The maximum removal percentage of TKN, BOD, and COD before secondary treatment were conducted using mixture of TiO_2_ NPs, FC, and chitosan, which reached 44.2, 44 and 46.3%, respectively. The maximum removal percentage of TKN, BOD, and COD after secondary treatment were conducted using mixture of TiO_2_ NPs, FC, and chitosan, which reached 94.9, 99.7 and 99.6%, respectively. Overall, the results derived from this investigation suggest that the TiO_2_ NPs/UV holds significant advanced treatment of sewage water, making it a viable choice for water reuse applications.

## Introduction

The continued social and economic development and population growth are increasing pressure on the world's water resources, facing the challenges of water scarcity and deteriorating water quality^1–3^. Water scarcity is a serious issue that affects both humankind's way of life and the global economy^4–6^. One of the potential solutions to deal with water scarcity is utilizing non-conventional water resources^7^. Currently, emerging contaminants (ECs) have been detected in diverse effluents of surface water and treated municipal wastewater. ECs include pesticides, pharmaceuticals, and personal care and household items^8^ which, should be removed due to their toxicity, and other undesirable qualities^9,10^. Using the advanced oxidation processes (AOPs) is sufficient for these non-degradable pollutants in the wastewater drains that cannot be destroyed by conventional treatment^9,11–13^. AOPs, which depend on the non-selective reaction of hydroxyl radicals (^**⋅**^OH), are effective methods to degrade organic contaminant^14,15^. Heterogeneous AOPs include catalytic ozonation, photocatalytic ozonation, and heterogeneous photocatalysis^16,17^. The effective photocatalyst must be chemically and biologically inert, non-toxic, photoactive, economical, photostable, and able to use visible or near UV light. Catalysts include Si, WO_3_, ZnO, CdS, TiO_2_, ZnS, Fe_2_O_3_, SnO_2_, etc.^18^. Titanium dioxide (TiO_2_) is a commonly used catalyst due to its distinctive optical features, non-toxicity, low cost, and great photochemical stability^19–22^. There are various sources for titanium dioxide which is found in the form of ilmenite (40–80% TiO_2_) and mineral sand deposits such as anatase (> 95%TiO_2_), rutile (~ 95% TiO_2_) and leucoxene (> 65% TiO_2_)^23^. It’s important to create high-purity titanium dioxide from ilmenite by developing appropriate techniques with little environmental impact^24^. There are two established methods to produce titanium dioxide from ores: the chloride process and the sulfate process^24,25^. The chloride process is more efficient than sulphate method^23,26^. There is an important need to discover alternative techniques to extract high-grade TiO_2_ from titanium ores e.g., ilmenite which is available in large quantities in nature.

The goal of this paper is to extract titanium dioxide (TiO_2_) from ilmenite by different leaching agents such as: (1) HCl, HNO_3_, and Aqua Regia (mixture of nitric acid and hydrochloric acid) solutions. (2) Determine the optimal conditions for high-grade TiO_2_ extraction. (3) Characterization of raw ilmenite, intermediate materials and extracted titanium dioxide using different analytical techniques. (4) Conversion of titanium dioxide to titanium dioxide nano particle (TiO_2_NPs). (5) Chemical treatment of sewage wastewater using ferric chloride which is a byproduct of extracted titanium dioxide (6) utilization of TiO_2_ for removal of organics.

## Materials and methods

### Materials

Ilmenite samples were collected from the western side of Wadi Abu Ghalaga, South Eastern Desert of Egypt. Humic acid, hydrochloric acid (HCl) and nitric acid (HNO_3_) were obtained from the Fisher Scientific Company. Aqua regia solution was prepared in the laboratory (HNO_3_ and HCl, 1:3). Double distilled water was used. Dissolution experiments of ilmenite ore were conducted in rounded flasks attached to a condenser and stirrer.

### Experimental procedure

**Figure 1:**
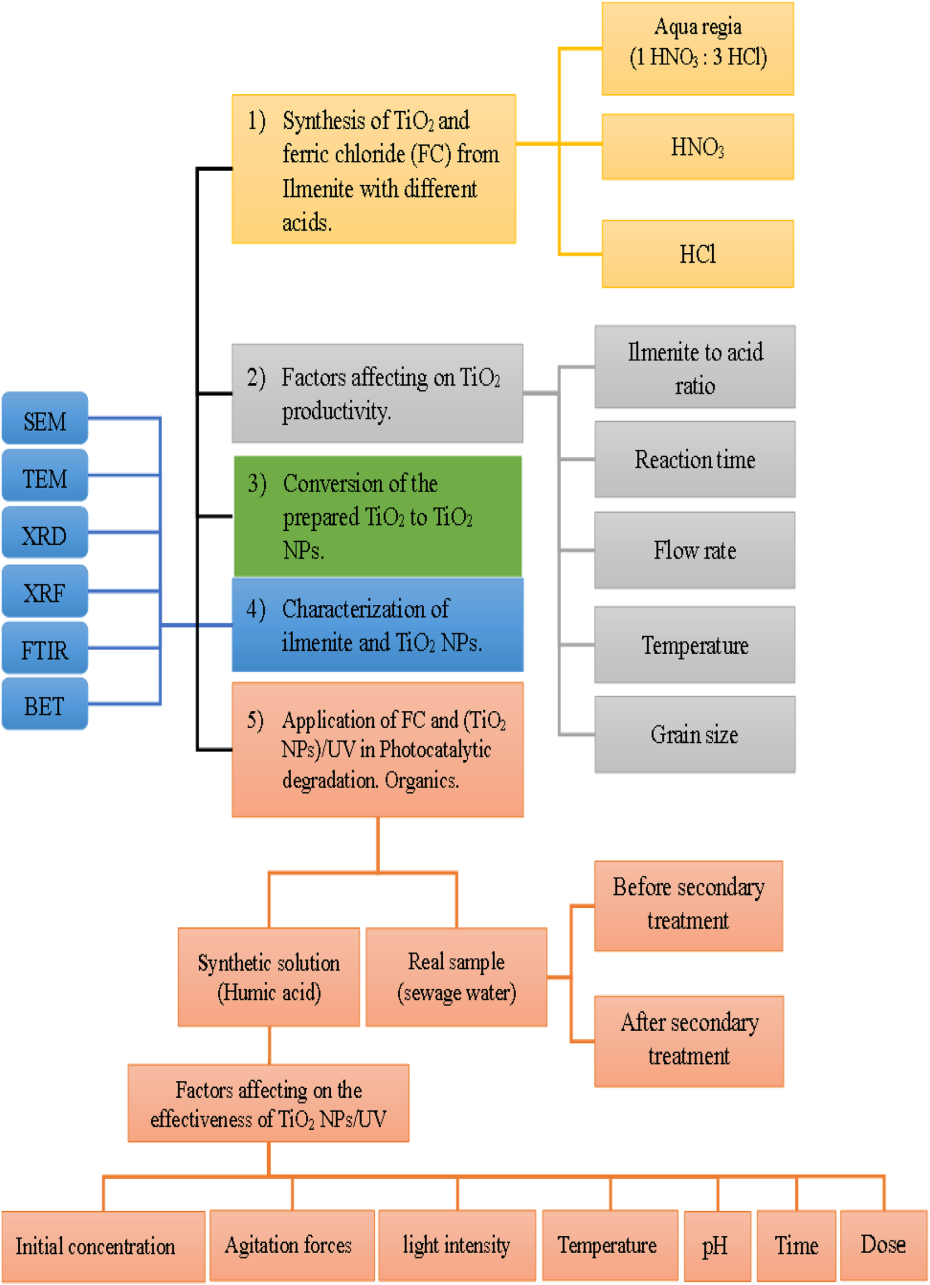
The systematic diagram of all experimental procedures.

As shown in Fig. 1 the systematic diagram of all experimental procedures is explored.

#### Leaching titanium dioxide and ferric chloride (FC) from ilmenite

Ilmenite samples were crushed and ground by using a grinding machine. The crushed samples were sieved through a 200-mesh sieve (75 µm). The ilmenite sample leached with acids under several conditions. After each leaching experiment, the product was filtered, and rinsed with distilled water, the filtrate called FC. Several affecting factors were studied including acids ratio, grain size, temperature, flow rate (amount of acid add (ml) per time(minute), and reaction time as follows:

##### Effect of acid ratio

The ratio of 1:1, 1:2.1:31:6 at a constant (flow rate (0.5 ml/min), time of reaction (3 h) ilmenite particle size 75 micron, and reaction temperature 120 °C.

##### Effect of reaction time

30, 60, 120, 180, 240 min were chosen at constant reaction time 3 h., flow rate (0.5 ml/min), ilmenite particle size 75-micron, ilmenite acid ratio (1:3) and reaction temperature 120 °C.

##### Effect of flow rate

0.5, 1, 3, and 9 ml/min were chosen as different follow rates with a constant (time of reaction is (3 h), ilmenite particle size 75-μm, ilmenite acid ratio (1:3) and reaction temperature 120 μC.

##### Effect of temperature

80, 100, 110, and 120 μC at a constant (flow rate (0.5 ml/min), time of reaction (3 h) ilmenite particle size 75-μm, and ilmenite acid ratio (1:3).

##### Effect of particle size of ilmenite

300, 200, 100, and 75 microns at a constant (flow rate (0.5 ml/min), time of reaction (3 h), reaction temperature 120 °C, and ilmenite acid ratio (1:3).

#### Conversion of the prepared titanium dioxide to titanium dioxide nano particles (TiO_2_ NPs)

5 g of the prepared titanium dioxide react with suitable acid with molar ratio (3:1), add 100 m distilled water, the product neutralize with ammonia (2 M) until pH 11, stirring, and heating at 90 °C for 30 min, filter the mixture, wash by distilled water, drying at 105 °C for 2 h., calcination at 550 °C, the product named titanium dioxide nano particles (TiO_2_ NPs).

#### Characterization of ilmenite and titanium dioxide nano particles (TiO_2_ NPs)

Characterization of raw ilmenite and titanium dioxide nano particles (TiO_2_ NPs) were carried out by using different analytical techniques such as transmission electron microscopy (TEM) using a Zeiss EM-90 operating at 80 kV tension. Scanning Electron Microscope (SEM) Model Jeol 6510 JSM, LA. Brunauer–Emmett–Teller (BET) by using N2 adsorption/desorption at 77 K using an automatic surface area device (BELSORP MINI X). X-ray diffraction (XRD) (Paralytical Philips APD-3720, Netherlands) with Cu–kα radiation (λ = 0.154 cm^−1^) and operated at 40 kV, 35 mA, 5 min scanning speed in the 2θ range of 5°–80°. Fourier transform infrared (FTIR) spectrum of TiO_2_ NPs was recorded in the range of 400–4000 cm^−1^ with a Bruker FT/IR-2000 spectrometer. X-ray fluorescence (XRF) technique using Axios MAX, PAN analytical, 40 kV, 50 Ma.

#### Application of (TiO_2_ NPs)/UV for removal of humic acid from synthetic solution

The humic acid of 50 mg/l was prepared, the effectiveness of TiO_2_ NPs/UV for photocatalytic degradation of humic acid was carried out, the affecting factors such as dose, light intensity, pH, initial concentration, and agitation forces were conducted.

#### Application of FC and TiO_2_ NPs/UV for real sample of sewage water treatment

A certain sample of sewage water before and after secondary treatment was treated using a binary system of ferric chloride and titanium dioxide nano particles (TiO_2_ NPs) for advanced treatment of sewage water. The main characteristics of sewage water such as COD, BOD, TSS, and TKN are analyzed according to standards methods^27^.

#### Utilization of the prepared FC in chemical treatment for sewage water

The sample of the prepared ferric chloride (FC) coagulant was used to remove some pollutants such as BOD, COD and TSS from sewage water where the treatment depends on precipitation, coagulation, and adsorption techniques by poly inorganic coagulants (PIC). Each sample was mixed with 8–13 ppm of PIC and agitated for 1 min rapid mixing (200 rpm), followed by slow mixing for five minutes and (40 rpm) and 30 min standing time. The concentrations of pollutants were measured in ore samples and in the filtrate according to standards methods^27^.

#### Utilization of the prepared TiO_2_ NPs/UV in treatment of sewage water

The sample of the prepared TiO_2_ NPs was used to remove some pollutants such as BOD, COD, TKN, TOC and TSS from sewage water where the treatment depends on photocatalytic degradation. Each sample was mixed with 8–13 ppm of TiO_2_ NPs/UV and agitated for 30 min rapid mixing (200 rpm), followed by standing time for 30 min. The concentrations of pollutants were measured in ore samples and in the filtrate in Eq. (1) according to standards methods^27^.

1 $$\mathbf{R}\mathbf{\%}=\frac{{\mathbf{C}}_{0}-{\mathbf{C}}_{\mathbf{e}}}{{\mathbf{C}}_{0}}\times 100$$

where C_0_ and Ce are the initial and final concentrations (mg/l); respectively.

## Results and discussion

### Factors affecting the productivity of TiO_2_

#### Effect of reaction temperature

**Figure 2:**
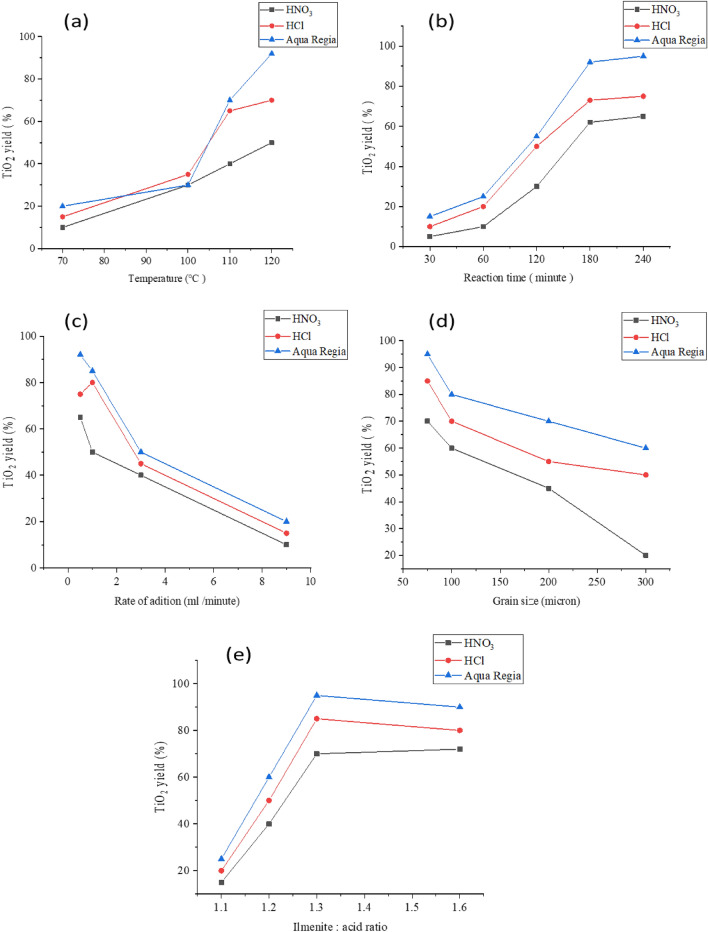
(**a**) Effect of temperature on TiO_2_ productivity, (**b**) effect of reaction time on TiO_2_ yield, (**c**) effect of rate of addition on TiO_2_ yield, (**d**) effect of grain size (micron) on TiO_2_ yield, (**e**) effect of ilmenite: acid ratio on TiO_2_ yield.

The endothermic nature of the reaction is clearly indicated Fig. 2a. The increase of temperature raises the production of TiO_2_. The result showed that as temperature increases the produced percentage of TiO_2_ increases. The optimum production condition is at 160 °C, the elevated temperature was required to overcome the binding between different metal oxides in ilmenites ore material.

#### Effect of reaction time

The effect of the contact time on the percentage of the produced TiO_2_ was shown in Fig. 2b. The results revealed that as the contact time increases, the TiO_2_ production increases where the upper productivity limit was at time 3 h of direct contact between the reagents. The long time for reaction was required also to overcome the binding between ingredients of ore material, which compatible with effect of temperature.

#### Effect of rate of addition

The flow rate has a significant effect on the production of TiO_2_ Fig. 2c. The result showed that as flow rates increase the produced TiO_2_ decreases. It became clear from the results that the best flow rate to obtain the highest production rate is 0.5 ml/min. The slow rate of addition gave the chance for formation product easily.

#### Effect of ilmenite grain size

The large, exposed surface area of the ilmenite enhances the reaction and then the production of TiO_2_ increases Fig. 2d. The result showed that as the grain size increases the production of TiO_2_ decreases where the best size for the optimum condition is 75 μm. The small size of ilmenite increases the rate of reaction due to the increase of surface area.

#### Effect of ilmenite:acid ratio

The relation between the ilmenite and acids ratio and the percentage of the produced TiO_2_ was illustrated in Fig. 2e. The result showed that ratio increases the productivity content of TiO_2_ increases. The optimum productivity limit was 1:3. which prof that stochiometric calculations are compatible with empirical formula.

From the results the optimum conditions of TiO_2_ as follows: time of reaction (3 h), temperature (160 °C), rate of reaction (1 ml/min), ilmenite acid ratio (1:3), and ilmenite grain size 75 μm.

The higher productivity was obtained using aqua regia due to double action of hydrochloric acid and nitric acid.

### Characterization of materials

#### TEM and SEM characterizations

**Figure 3:**
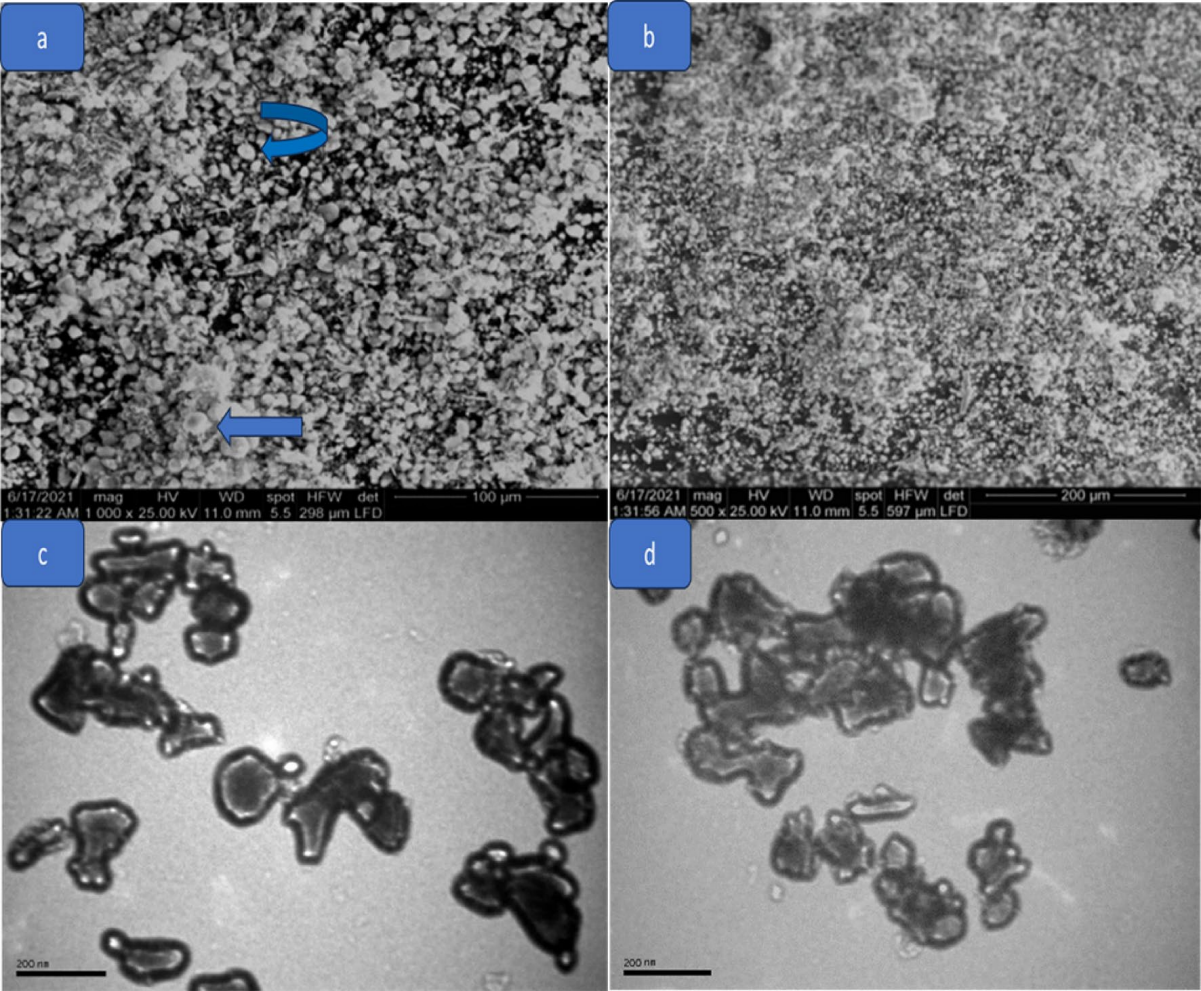
(**a**, **b**) SEM and (**c**, **d**) TEM images of TiO_2_ NPs.

Scanning electron microscopy (SEM) was employed to examine the surface structure of TiO_2_ nanoparticles. Figure 3a,b shows the SEM image of synthesized TiO_2_ NPs, which clearly revealed their spherical shape with very little aggregation. Additionally, it was noted that all the particles were in the nanoscale range^28–34^.

TEM analysis provided information about the shape and size of TiO_2_ NPs. Figure 3c,d represents the TEM micrographs of synthesized TiO_2_ NPs. The result showed that the TiO_2_ NPs have an average particle size of 92 nm. The TiO_2_ nanoparticles have spherical and elliptical shapes with agglomerated morphology^35^.

#### Surface area characterization

**Figure 4:**
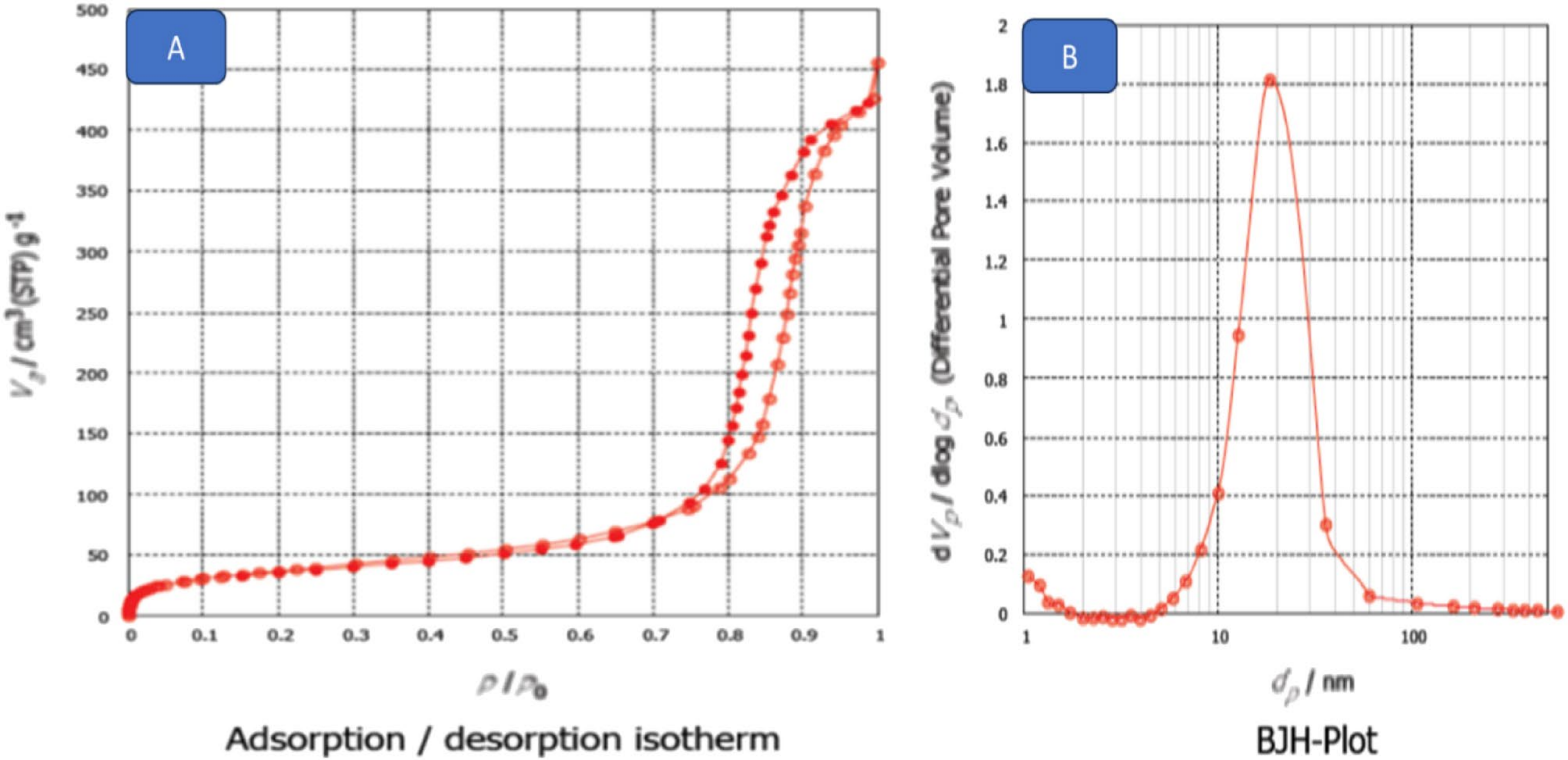
(**A**) Adsorption–desorption isotherm, (**B**) BJH curve figure of titanium dioxide nano particles (TiO_2_ NPs).

The surface area, pore volume, and porosity of TiO_2_ nanoparticles were assessed using nitrogen adsorption/desorption measurements, specifically the Brunauer–Emmett–Teller (BET) method, as depicted in Fig. 4A and Table 1. The results indicated a Type II isotherm with a small H1 hysteresis loop, as per the IUPAC classification. In Figure A, the pore characteristics of TiO_2_ nanoparticles were observed to be in the meso/micro range, with a monolayer capacity (Vm) of 2.0 cm^3^/g and an average pore diameter of 28.29 nm. The surface area (SBET) for the nano TiO_2_ was measured at 88.15 m^2^/g, and the total pore volumes (Vp) were found to be 0.0616 cm^3^/g. To analyze the pore size distribution, the Barrett–Joyner–Halenda (BJH) method was applied, as presented in Fig. 4B and Table 1. The BJH curve revealed an average pore size of 40 nm, indicating that the majority of the nanoparticle pores are mesopores^28,29,33,36^.

**Table 1:** Porosity and surface area of TiO_2_NPs.

Sample	BJH adsorption cumulative pore volume (cm^3^/g) (nm)	Pore size (nm)	BET surface area (m^2^/g)
TiO_2_	0.0616	28.29	88.15

#### XRD characterization

**Figure 5:**
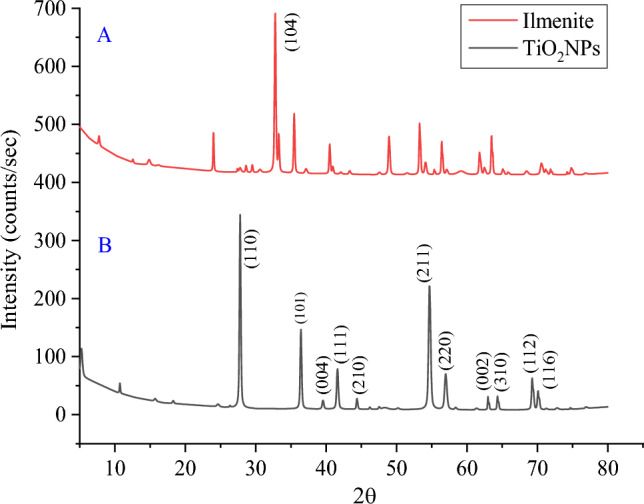
(**A**) XRD of ilmenite, (**B**) XRD of titanium dioxide nano particles (TiO_2_ NPs).

The XRD spectra of ilmenite ore and the synthesized TiO_2_ NPs is shown in Fig. 5. The XRD analysis as shown in Fig. 5A revealed the presence of two mineral phases: ilmenite and hematite. the ore is primarily composed of ferri-ilmenite with trace amounts of titano-hematite. The presence of the (104) peak at 2θ = 32.78 demonstrated the presence of ilmenite in the XRD pattern^37–39^.

Figure 5B shows the sharp peaks appearance for TiO_2_ NPs at 2θ values 27.78°, 3642°, 39.54°, 41.62°, 44.38°, 54.66°, 56.98°, 63.14°, 64.34°, 69.26°, and 70.1° at corresponding Miller indices (110), (101) (004), (111), (210), (211), (220), (002), (310), (112) and (116) respectively, confirms the formation of highly crystalline TiO_2_ NPs^28,29,31,36,40^.

#### FTIR characterization

**Figure 6:**
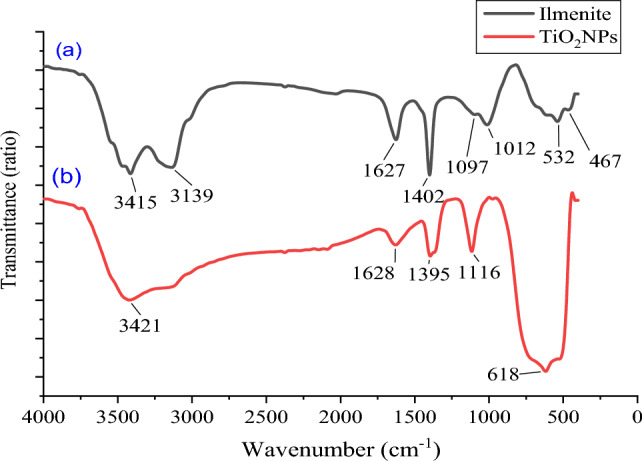
(**a**) FTIR of ilmenite, (**b**) FTIR of titanium dioxide nano particles (TiO_2_ NPs).

The FT-IR spectrum of Ilmenite is shown in Fig. 6a. The 467 cm^−1^ and 532 cm^−1^ bands in the ilmenite spectra were linked to the Fe–O bonding, which is the distinctive band of ilmenite. The bending mode of adsorbed water on the ilmenite surface was assigned to the band of 3415 cm^−1^^38,41,42^.

The FT-IR spectrum of synthesized TiO_2_ nanoparticles is shown in Fig. 6b. Within this spectrum, the absorption peak at 3452.2 cm^−1^ corresponds to the –OH stretching, while the peak at 1635.5 cm^−1^ is associated with the –OH bending vibration, which indicates the presence of water as moisture. Moreover, the strong peak at 690.5 cm^−1^ is attributed to the Ti–O stretching band, which is a distinctive characteristic of TiO_2_^28,29,33,43–45^.

#### XRF characterization

As shown in Table 2 the elemental analysis of ilmenite and Titanium dioxide nano particles (TiO2 NPs) explored by XRF, the main contents of metal oxides of ilmenite such as SiO_2_, Al_2_O_3_, Fe_2_O_3_, MgO, SO_3_, K_2_O, Na_2_O, Cr, MnO_2_ and TiO_2_ is 14.07, 3.75, 34.2, 4.88, 1.95, 0.08, 0.19, 1526, 1.63 and 32.72 respectively and loss of ignition is 6.3774 and any variation in chemical composition is due to different sources localities^46,47^. Whereas the major elemental oxide of TiO_2_ nanoparticles is 95.6 and loss of ignition is 4.4^35,47^.

**Table 2:** The elemental analysis of ilmenite and titanium dioxide nano particles (TiO_2_ NPs).

Sample	SiO_2_	Al_2_O_3_	Fe_2_O_3_	MgO	K_2_O	Na_2_O	Cr	MnO_2_	TiO_2_	LOI
Ilmenite %	14.07	3.75	34.2	4.88	0.08	0.19	0.15	1.63	32.72	6.38
TiO_2_ NPs%	0.01	0.02	0.1	0.03	0.11	0.23	0.001	0.023	95.6	3.89

### Affecting factors on the effectiveness of TiO2 NPs/UV for photocatalytic degradation of humic acid

#### Effect of dose

**Figure 7:**
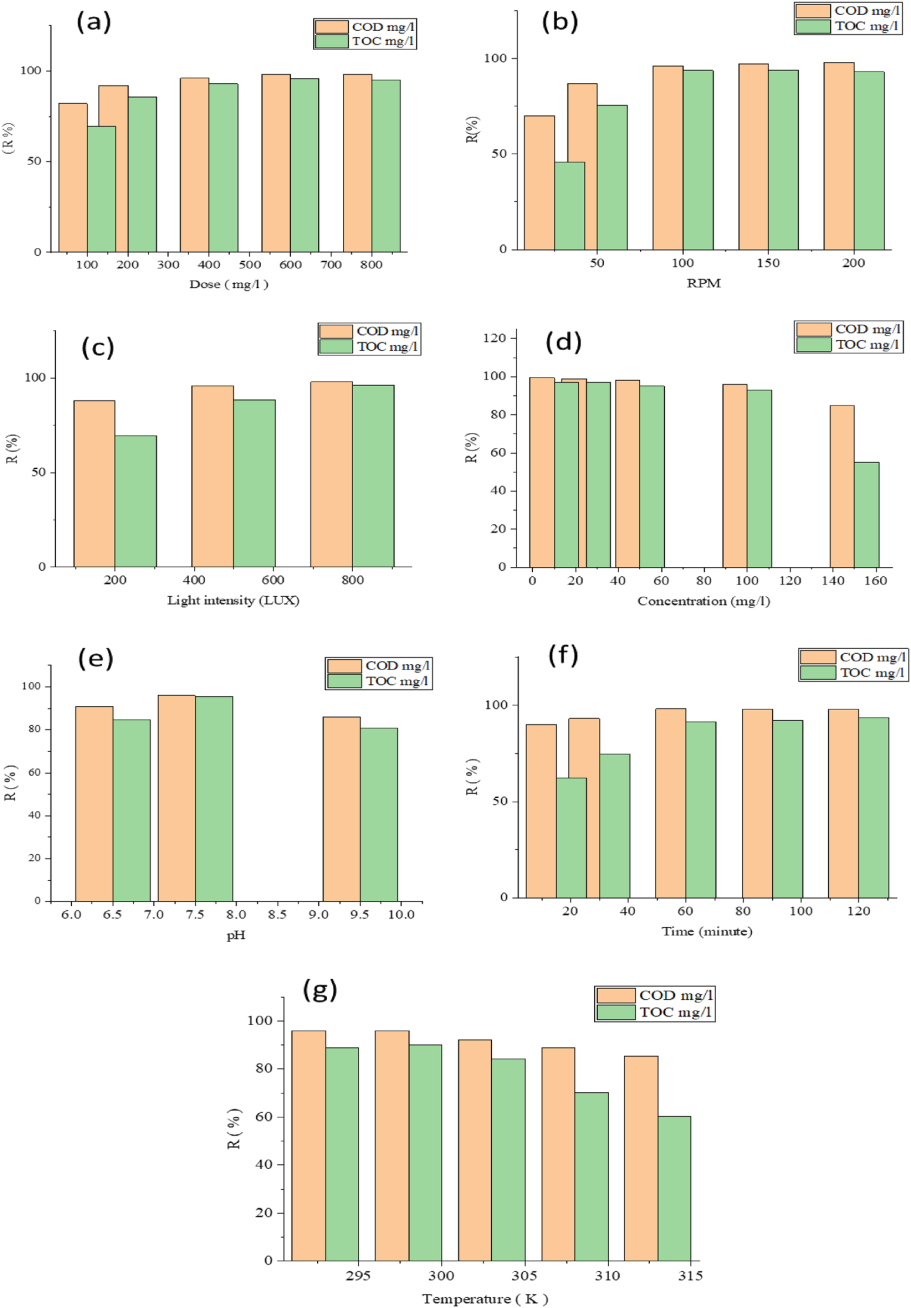
Variation of COD and TOC R% versus (**a**) dose(mg/l), (**b**) RPM, (**c**) light intensity (LUX), (**d**) concentration (mg/l), (**e**) pH, (**f**) reaction time (min), (**g**) temperature degree (K) of TiO_2_ NPs.

The relation between the dose and the percentage of the removal of TOC and COD at constant (agitation force 200 rpm, light intensity 500 lx, contact time 60 min, pH 7.5, temperature 298 K and Concentration 100 mg/l) was illustrated in Fig. 7a. The highest removal percentages of COD and TOC recorded at dose 600 mg/l. whereas the highest removal percentage which recorded by Mohammed et al. was 4 g/l^48–50^, Aljuboury et al. was 0.5 g/l^51^, Sirisha et al. was at a dosage of 1.5 g/l^52^. Also, Joy et al. was 0.51 g/l^53^ and Surendra et al. recorded that the highest removal percentage was at a dosage of 1 g/l^54^.

#### Effect of agitation forces

The relation between the RPM and the percentage of the removal of TOC and COD at constant (dose 400 mg/l, light intensity 500 lx, contact time 60 min, pH 7.5, temperature 298 K and concentration 100 mg/l) was illustrated in Fig. 7b. The highest removal percentages of COD and TOC recorded at 200 rpm for 60 min of spinning. whereas the highest removal percentage which was recorded by Surendra et al. was at 600 rpm for 60 min of spinning^54^.

#### Effect of light intensity

The relation between the light intensity and the percentage of the removal of TOC and COD at constant (dose 400 mg/l, agitation forces 100 rpm, contact time 60 min, pH 7.5, temperature 298 K and concentration 100 mg/l) was illustrated in Fig. 7c. The highest removal percentages of COD and TOC recorded at light intensity 800 lx. whereas the highest removal percentage which recorded at light intensity 3.0 mw/cm^2^^55–57^.

#### Effect of initial concentration

The relation between concentration of TiO_2_ and the percentage of the removal of TOC and COD at constant (dose 400 mg/l, agitation forces 100 rpm, contact time 60 min, pH 7.5, temperature 298 K and light intensity 500 lx) was illustrated in Fig. 7d. The highest removal percentages of COD and TOC recorded at concentration 10 mg/l. whereas the highest removal percentage which recorded by Sirisha et al. was 5 mg/l^52^. Also, Surendra et al. recorded that the highest removal percentage was at initial concentration of 20 mg/l^54^.

#### Effect of pH

The relation between pH and the percentage of the removal of TOC and COD at constant (dose 400 mg/l, agitation forces 100 rpm, contact time 60 min, light intensity 500 lx, temperature 298 K and concentration 100 mg/l) was illustrated in Fig. 7e. The highest removal percentages of COD and TOC were recorded at pH 7.5. whereas the highest removal percentage which recorded by Mohammed et al. was at pH 7.5^48^ and Sirisha et al. (92.41%) was at pH of 8^52^. Also, Surendra et al. recorded that the highest removal percentage was at pH of 7^54^.

#### Effect of time

The relation between time and the percentage of the removal of TOC and COD at constant (dose 400 mg/l, agitation forces 100 rpm, pH 7.5, light intensity 500 lx, temperature 298 K and concentration 100 mg/l) was illustrated in Fig. 7f. The highest removal percentages of COD and TOC recorded at time 90 min. whereas the highest removal percentage which recorded by Mohammed et al. was 180 min^48,49^, Aljuboury et al. was 170 min^51^, and Surendra et al. recorded that the highest removal percentage was at 60 min^54^.

#### Effect of temperature

The relation between degree of temperature and the percentage of the removal of TOC and COD at constant (dose 400 mg/l, agitation forces 100 rpm, pH 7.5, light intensity 500 lx, contact time 60 min and concentration 100 mg/l) was illustrated in Fig. 7g. The highest removal percentages of COD and TOC recorded at temperature 298 (K). whereas the highest removal percentage which recorded by Sirisha et al. was 333 K^52^. Also, Surendra et al. recorded that the highest removal percentage was at temperature of 70 °C^54^.

### Application of binary system on real sample of sewage water treatment

From Table 3, the removal percentages of TKN, BOD, COD and TSS before secondary treatment using FC are 32, 29, 34 and 97.3, respectively. The removal percentages of TKN, BOD, COD and TSS before secondary treatment using TiO_2_ NPs/UV are 93.5, 99.5, 99.6 and 84.7, respectively. The removal percentages of TKN, BOD, COD and TSS before secondary treatment using TiO_2_ NPs/UV and FC are 94.7, 99.7, 99.7 and 85.5, respectively. The removal percentages of TKN, BOD, COD and TSS before secondary treatment using TiO_2_ NPs/UV, Fc and chitosan are 94.9, 99.7, 99.6 and 87.2, respectively.

**Table 3:** Studies of removal percentages of TKN. BOD, COD and TSS before secondary treatment using different chemical reagents.

Dose	TKN (mg/l)		BOD (mg/l)		COD (mg/l)		TSS (mg/l)	
	Initial	Final	Initial	Final	Initial	Final	Initial	Final
0.01 g of FeCl_3_	177.4	120.1	1057	750	1410	930	1114.5	30
Removal %	32		29		34		97.3	
0.1 g of TiO_2_ (NPs)	177.4	11.5	1057	4.5	1410	5.5	1114.5	170
Removal %	93.5		99.5		99.6		84.7	
0.1 g TiO_2_ (NPs) + FeCl_3_	177.4	9.4338	1057	3.3	1410	4.44	1114.5	162
Removal %	94.7		99.7		99.7		85.5	
0.1 g TiO_2_ (NPs) + 10 ml FeCl_3_ + 2 ml chitosan	177.4	8.9	1057	3.4	1410	4.56	1114.5	142
Removal %	94.9		99.7		99.6		87.2	

From Table 4, the removal percentages of TKN. BOD, COD and TSS after secondary treatment using FC are14.8, 9.9, 13.2 and 96.9, respectively. The removal percentages of TKN. BOD, COD and TSS before secondary treatment using TiO_2_ NPs/UV are15.4, 14.2, 22.3 and 69.8, respectively. The removal percentages of TKN. BOD, COD and TSS before secondary treatment using TiO_2_ NPs/UV and FC are 33.8, 31.8, 33.3 and 45.5, respectively. The removal percentages of TKN. BOD, COD and TSS before secondary treatment using TiO_2_ NPs/UV, FC and chitosan are 44.2, 44, 46.3 and 54.5, respectively. The maximum percentages removal of COD, BOD and TSS in sewage wastewater reached 90, 92, and 93%, respectively^58^, the maximum removals of TSS, COD and BOD are 94.2, 89.2, and 76.9% respectively using PAlFeClSi^59^. the ultimate removal percentages of TSS, COD and BOD were 92.0, 89.0, and 91.0%, respectively by PAlFeCl + Si^60^.

**Table 4:** Studies of removal percentages of TKN. BOD, COD and TSS After secondary treatment using different chemical reagents.

Dose	TKN (mg/l)		BOD (mg/l)		COD (mg/l)		TSS (mg/l)	
	Initial	Final	Initial	Final	Initial	Final	Initial	Final
0.01 g of FeCl_3_	65.4	55.7	9.1	8.2	12.1	10.5	166.5	5.1
Removal %	14.8		9.9		13.2		96.9	
0.1 g of TiO_2_ (NPs)	65.4	55.3	9.1	7.8	12.1	9.4	166.5	50.2
Removal %	15.4		14.2		22.3		69.8	
0.1 g TiO_2_ (NPs) + 30 ml FeCl_3_	65.4	43.3	9.1	6.2	12.1	8.2	166.5	90.6
Removal %	33.8		31.8		33.3		45.5	
0.1 g TiO_2_ (NPs) + 30 ml FeCl_3_ + 2 ml chitosan	65.4	35.2	9.1	5.1	12.1	6.5	166.5	76.5
Removal %	44.2		44		46.3		54.5	

## Conclusion

This paper investigated the leaching of ilmenite to extract titanium dioxide via utilizing leaching procedures with various ingredients such as hydrochloric acid, nitric acid, and Aqua Regia then application of titanium dioxide plus ultraviolet radiation in advanced wastewater treatment. The affecting factors on titanium dioxide extraction such as ilmenite to acid ratio, reaction time, ilmenite grain size, rate of addition and reaction temperature were conducted. The best leaching conditions obtained were Ilmenite to acid ratio: 1:3 respectively, time: 3 h, grain size: 75 μm, temperature: 160 °C, rate of addition: 0.5 ml/min. Titanium dioxide nano particles (TiO_2_ NPs) were prepared. Comprehensive physico-chemical characterization of Ilmenite and TiO_2_ NPs were characterized using different analytical techniques. All the analytical techniques proved the formation of titanium dioxide and titanium dioxide nanoparticles. Affecting factors on the effectiveness of TiO_2_ NPs for photocatalytic degradation such as dose, agitation forces, light intensity, initial concentration, pH, time, and temperature are conducted. The optimum conditions for TiO_2_ NPs/UV photocatalytic degradation obtained were dose 600 mg/l, agitation force 200 rpm for 60 min of spinning, light intensity 800 lx, initial concentration 10 mg/l, pH 7.5, time 90 min and temperature 298 (K). The removal percentages of Total suspended solids (TSS), chemical oxygen demands (COD), biological oxygen demand (BOD)and total nitrogen (TN) were explored. According to results, the maximum removal percentage of TSS were 97.3 and 96.9% before and after secondary treatment which were conducted using FC. The maximum removal percentage of TKN, BOD, and COD before secondary treatment were conducted using mixture of TiO_2_ NPs, FC, and chitosan, which reached 44.2, 44 and 46.3%, respectively. The maximum removal percentage of TKN, BOD, and COD after secondary treatment were conducted using mixture of TiO_2_ NPs, FC, and chitosan, which reached 94.9, 99.7 and 99.6%, respectively. All in all, the results derived from this investigation suggest that the TiO_2_ NPs/UV holds significant promise for effective advanced treatment of sewage water, making it a viable and appropriate choice for water reuse applications.

## Data Availability

All relevant data are included in the paper.

## Abbreviations

ECs: Emerging contaminants
AOPs: Advanced oxidation processes
APHA 2022: American Public Health Association 2022
BET: Brunauer–Emmett–Teller
BJH: Barrett–Joyner–Halenda
BOD: Biological oxygen demand
COD: Chemical oxygen demand
FC: Ferric chloride
FTIR: Fourier transform infra-red
LOI: Loss of ignition
SEM: Scanning electron microscopy
TEM: Transmittance electron microscopy
TiO_2_ NPs: Titanium dioxide nanoparticles
TKN: Total Kjeldahl nitrogen
TSS: Total suspended solids
XRF: X-ray fluorescence
